# From eye movements to scanpath networks: A method for studying individual differences in expository text reading

**DOI:** 10.3758/s13428-022-01842-3

**Published:** 2022-04-20

**Authors:** Xiaochuan Ma, Yikang Liu, Roy Clariana, Chanyuan Gu, Ping Li

**Affiliations:** 1 Department of Psychology, The Pennsylvania State University, Moore Building, University Park, PA 16802 USA; 2 Department of Biomedical Engineering, The Pennsylvania State University, Millennium Science Complex, University Park, PA 16802 USA; 3 Department of Learning and Performance Systems, Keller Building, The Pennsylvania State University, University Park, PA 16802 USA; 4 Department of Chinese and Bilingual Studies, Faculty of Humanities, The Hong Kong Polytechnic University, Hung Hom, Kowloon, Hong Kong

**Keywords:** Reading comprehension, Eye tracking, Knowledge representation, Scanpath, Network metrics

## Abstract

Eye movements have been examined as an index of attention and comprehension during reading in the literature for over 30 years. Although eye-movement measurements are acknowledged as reliable indicators of readers’ comprehension skill, few studies have analyzed eye-movement patterns using network science. In this study, we offer a new approach to analyze eye-movement data. Specifically, we recorded visual scanpaths when participants were reading expository science text, and used these to construct scanpath networks that reflect readers’ processing of the text. Results showed that low ability and high ability readers’ scanpath networks exhibited distinctive properties, which are reflected in different network metrics including density, centrality, small-worldness, transitivity, and global efficiency. Such patterns provide a new way to show how skilled readers, as compared with less skilled readers, process information more efficiently. Implications of our analyses are discussed in light of current theories of reading comprehension.

## Introduction

A significant amount of knowledge in both formal and informal settings is acquired through reading. In school, learners’ ability to efficiently integrate and obtain information during reading of scientific texts contributes significantly to their learning outcomes. The processes of reading include letter identification, lexical access, syntactic processing, and semantic integration (Perfetti & Stafura, 2014). The abilities involved in carrying out these processes constitute reading competency, often referred to as reading skill (Perfetti, 2001). In school settings, students’ reading skill is commonly assessed by post-reading comprehension questions. However, a single score derived this way is often insufficient to provide an accurate evaluation of the student’s reading skill (Dixon et al., 1988; Hasbrouck & Tindal, 2006; Perfetti, 1985). Readers differ from one another in terms of the order of reading, specifically the path, course, or trajectory during reading; for example, how they jump forward over some words or backtrack to others. In order to understand such individual differences during reading, and to promote reading competency, it is necessary for us to consider the trajectory during reading and its relationship with reading outcomes.

### Situation model and reading comprehension

The classical theory has viewed text reading as a process of building up or constructing a “situation model,” which refers to a reader’s mental representation of encoded information (Kintsch, 2005; Tapiero, 2007; Zwaan & Radvansky, 1998). Specifically, this situation model is constructed while a reader’s understanding of words, clauses, and sentences converges into a mental state of affairs described in the text. Information captured during reading can be theoretically viewed as key concepts and relations among them, and successful comprehension is achieved by establishing proper connections between concepts that make up a meaningful and coherent situation model. From this viewpoint, skilled readers can be defined as those who are capable of constructing situation models effectively and accurately (August et al., 1984; Zwaan & Brown, 1996). Besides reading skills, one’s background knowledge also plays an important role because the construction of situation models requires the reader to link and integrate newly acquired information to prior knowledge (Kendeou & van den Broek, 2007; Ozuru et al., 2009; van den Broek et al., 1999). This view is often referred to as the “construction-integration” model of text reading (Kintsch, 1988). Such integration is especially important for reading scientific text on a specific topic, as one’s background knowledge and familiarity with the subject can greatly promote interpretation and incorporation of information acquired during reading.

Reading comprehension also varies across text types; for example, reading of expository texts differs from that of narrative texts in many significant ways (for a review, see van den Broek, 2010). Because narrative texts tend to focus on temporal sequences of events, the reader tends to identify the change of events, and to engage in the process of constructive mental simulations of events and perspective-taking (Berman & Slobin, 1994; Best et al., 2008; Hickmann, 2003). Expository texts, by contrast, involve abstract concepts, often having no plot or storyline, and the reader’s task is not to engage in mental simulation of events, but to identify the different possible relationships among the concepts. These relationships can come in the form of sequential, logical, or hierarchical connections among concepts, rather than just sequential unfolding of events as in narrative texts (Britton, 1994; Meyer et al., 1980; van den Broek, 2010). Therefore, successful expository text comprehension requires not only accurate understanding of word meaning, but also fundamental world knowledge and the ability to construct and integrate intended connections among concepts. These characteristics make comprehending expository texts more challenging than reading narrative stories and require more integration of background knowledge from the reader.

Whether readers can efficiently construct a structured mental representation of the complex relationships among concepts is highly indicative of their depth of understanding of an expository text. Successful reading comprehension can be reflected by a mental representation that captures the intended meaning of a text. In this study, we are interested in how eye-movement patterns can reflect the overall process of constructing mental representations. Eye gaze patterns can potentially provide a significant amount of information on the reader’s attention allocation and semantic integration, both of which allow us to investigate how the situation model is built in a reader’s mind during knowledge acquisition via reading. Furthermore, these gaze patterns may also provide insights into how newly acquired information is eventually structured in the reader’s mental representation.

### Eye movements and visual scanpath

Starting from the early 1970s, eye gaze patterns have been acknowledged as a reflection of the involvement of mental information processing. The rapid increase in the use of eye-tracking methodology has been based on the “eye-mind assumption” that posits a tight link between human eye gaze and the focus of attention (Just & Carpenter, 1980). However, this assumption has been challenged by several studies in the literature (e.g., Kliegl et al., 2006; Mitchell et al., 2008; Schindler & Lilienthal, 2019), although the exact nature of the eye-mind relationship remains debatable (Pulido, 2021; Sharafi et al., 2020; Strohmaier et al., 2020). Previous eye-tracking studies of text reading (Rayner, 1978, 1998) have established a number of informative eye-movement features, including fixation (50–1500 ms pause of visual gaze on a segment), saccade (quick movement between two phases of fixations in the same direction), and regression (backward saccade to a previously visited segment). In the past decades, many studies of reading comprehension used eye-movement features as a moment-to-moment indicator of information processing. For instance, a number of eye-movement studies have investigated readers with various levels of reading skill. These studies showed that compared with average readers, highly skilled readers, as compared with less skilled readers, tend to exhibit shorter fixations, higher skip rate, and fewer regressions (e.g., Ashby et al., 2005; Chace et al., 2005). Readers’ eye movements also reflect the relative difficulty of the text, showing that as text becomes more difficult, readers make longer fixations, fewer skips, and exhibit more regressions (e.g., Jacobson & Dodwell, 1979).

It was not uncommon for earlier reading studies to treat the text material as a single entity or to rely on eye movement features that do not reflect text characteristics. However, in recent years more fruitful approaches have been brought into the field as researchers started to consider text characteristics and to use more eye movement features. For example, Yeari et al. (2017) specifically investigated the effect of highlighting when it comes to reading central vs. peripheral information in expository texts. Results of the study suggested that both text highlighting and concept centrality can affect readers’ reinspection time but not initial processing time. For central information in the text, highlighting did not seem to affect readers’ processing and recall performance. By contrast, peripheral information tended to receive more revisits when highlighted. Based on these patterns, Yeari et al. (2017) inferred that skilled readers tended to prioritize the encoding of central information regardless of text highlighting. Thus, skilled readers distinguish between central and peripheral concepts while reading, perhaps because they could use prior knowledge and consider text structure. Ariasi et al. (2017) later examined the effect of text type (i.e., refutation text that challenges commonly held misconceptions) and sentence type on readers’ eye fixation measures. They further showed that refutation text could trigger reinspection for potential settlement of conceptual conflicts and therefore longer look-back duration. Sentence type also affected eye movements in that topic-introducing and topic-final sentences led to longer fixation time compared to topic-medial sentences, given that the former are usually the central concepts in the text. These studies advanced the field by examining text characteristics and treating text material not as a single entity, as was done in earlier studies, but as a complex domain involving multiple interconnected concepts.

Eye-movement features have also been applied to the study of reading to characterize reading strategy. Hyönä et al. (2002) focused specifically on fixation patterns and identified four different types of readers based on their reading strategies. These groups used different strategies that included (a) fast linear readers who have little regression, with fast processing speed, (b) slow linear readers who also have little reprocessing but with relatively slower speed, (c) nonselective readers who make many regressions, and (d) topic structure readers who make a significant amount of reinspection to headings, paying close attention to the text’s topic rather than to other less relevant peripheral terms. Hyönä et al. (2002) found that the topic structure readers performed the best on the reading comprehension task. This finding points to the importance of examining eye-movement trajectory based on reader characteristics and individual differences in reading strategies, other than measuring the overall eye-movement properties.

In this study we have adopted the “visual scanpath” as a useful measurement to understand the relationships between eye-movement patterns and text characteristics on one hand, and eye-movement trajectories and individual reading differences on the other. Visual scanpath refers to the record of the eye-movement trajectories when viewing and analyzing a visual stimulus, which consists of sequential fixations and saccades (Toh et al., 2011). We built visual scanpaths for this project based on real-time word-level fixation onsets, reflecting the actual reading input for each reader, specifically regarding which words their perceptual window is fixated on, and when. Based on the time point when a word is fixated, words in the original sentence will be alternately organized following one’s actual reading scanpath. For example, in reading “Could humans live on Mars some day?” the reader’s visual scanpath may be: “Could humans live Mars day Could humans.” This fixation-based word sequence may seem quite different from a complete sentence, but it reflects the actual word order that is fixated by the reader and thus where the reader’s attention is allocated over time.

Compared with well-investigated eye-movement features like average fixation duration, regression rate, skip rate, and so on, the use of scanpath as a useful cognitive indicator has only been recent (Luo et al., 2015; von der Malsburg et al., 2015). This is potentially due to the difficulty of quantifying an input in text format. In contrast to easily quantifiable general eye-movement features counting for ratio, count, angle, and time length in numeric format, scanpath data consist of essentially a collection of words. However, this collection of words contains rich structure and reflects mental processes that are not easily observable in other eye-movement features. In other research domains, visual scanpath has nevertheless been used, for example, in investigating students’ information processing during geometric text-figure integration (Lee & Wu, 2018), examining syntactic reanalysis of garden-path sentences (von der Malsburg & Vasishth, 2011), predicting participants’ performance on the Raven's advanced progressive matrices test (Hayes et al., 2011), and identifying expertise levels of dental students (Castner et al., 2018) and radiologists (Gandomkar et al., 2018) on X-ray reading, and predicting participants’ risk-taking behaviors (Zhou et al., 2016). These various studies have indicated the effectiveness and validity of visual scanpath as an index of information processing. To align reading research with advances in other domains, the current study makes use of the features in the scanpath data by adopting a network science approach, specifically to convert individual visual scanpaths into scanpath networks, which enables us to capture the rich structures embedded in visual scanpaths for individual readers.

### Scanpath networks of eye-tracking data

In recent years, network science has been applied to many fields, including cognitive science, as a tool to extract higher-order structures from large-scale data. Castro and Siew (2020) suggested that network science could model the structure of cognitive systems and dynamic processes within the systems by estimating the relationships among elements in the system such as words and concepts in memory. The networks that are derived from graph-theoretical analyses of big data suggest that the typology of nodes and edges, distance of nodes, communal structures, and general small-worldness can effectively capture social, linguistic, and developmental characteristics of human behavior. In addition, network measurements could reflect the structure of network representations at different levels (i.e., microscopic, mesoscopic, and macroscopic), providing more perspectives to explore the mechanisms behind human cognition (Siew et al., 2019).

The network science metrics could be applied to construct diverse cognitive networks in different research areas, such as semantic networks, form similarity networks, syntactic networks, and social networks (Siew et al., 2019). In these networks, definitions of nodes depicting representations and edges defining the relationship between these representations will depend on the specific cognitive phenomena being examined. For example, in the form similarity network, nodes are defined as words, and edges are defined as phonological or orthographic similarity between words. A higher coefficient indicates that more phonological or orthographic neighbors of a given word are also neighbors of each other, and longer reaction time and lower accuracy were observed for words with a high clustering coefficient than words with a low clustering coefficient in spoken word recognition task (Chan & Vitevitch, 2009). In semantic networks, nodes can be defined as words and edges as strengths of semantic similarity/connections among these words; a higher clustering coefficient indicates that more semantically similar words are clustered around a given node and therefore compete with one another during language production (e.g., less likely to be code-switches; Xu et al., 2021).

**Fig. 1:**
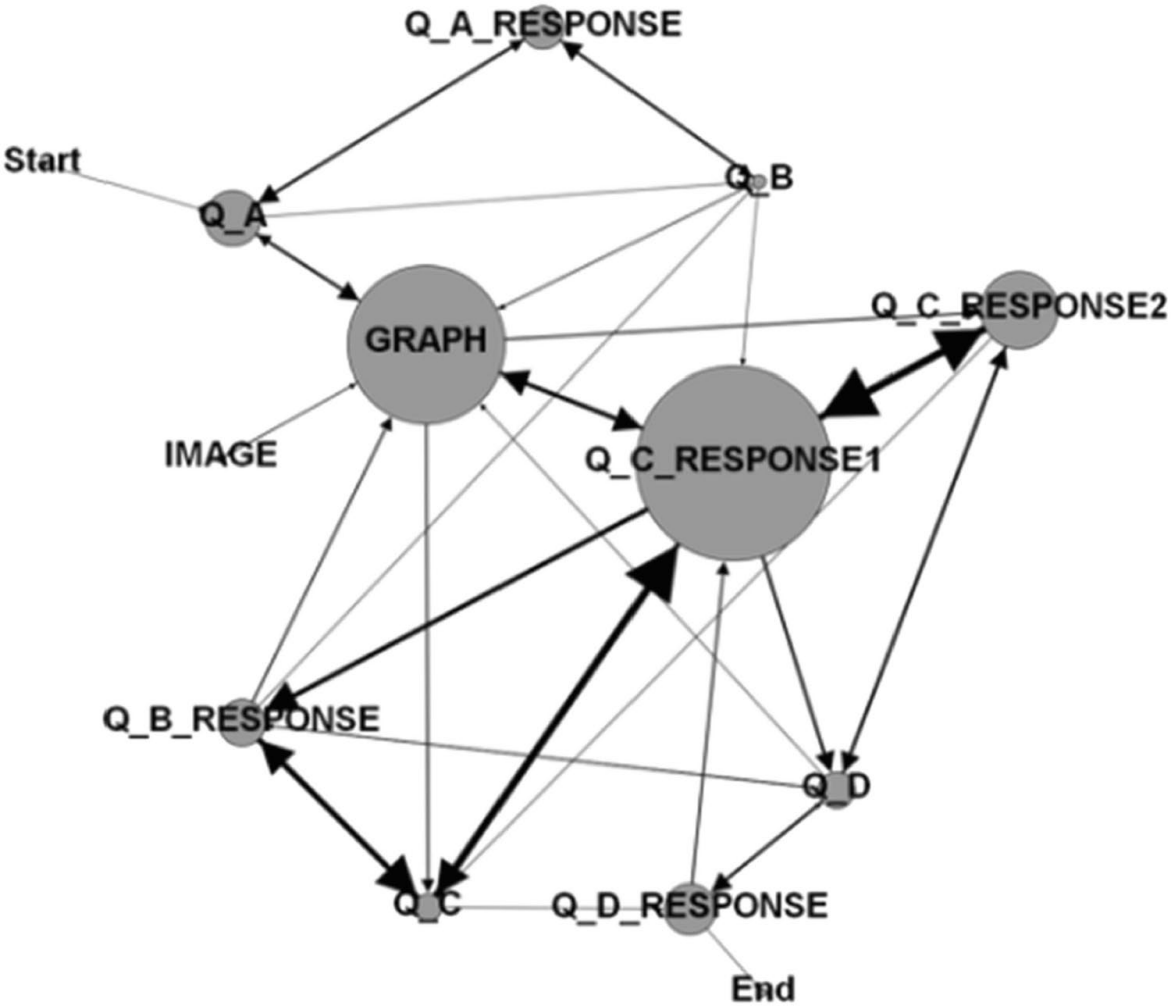
Example of visualization of one student’s transition network in Zhu & Feng’s study (2015). Nodes in the network represent AOIs, edges represent fixation transitions, and size of the nodes represent time spent in the AOI (bigger means longer). * *Permission of image reproduction granted by both authors*

Despite the utility of the network science approach, few studies have applied network metrics to analyze eye-movement data. One recent pilot study by Zhu and Feng (2015) combined these two to investigate mathematical problem-solving, and performed between-group comparison on an individual network level. The authors collected students’ gaze patterns when solving a math problem. They divided different screen areas into areas of interest (AOIs) based on where students’ eye gazes landed, and then constructed transition networks from their visual scanpaths by applying social network analytic methods (Fig. 1 illustrates how a network visualization may be constructed out of scanpath data). Inferences of participants’ problem-solving strategies were then made based on transition patterns in the networks: high-performing students exhibited more strategic fixation transitions, suggesting better abilities at connecting multiple information sources to solve complex problems, whereas low-performing students tended to consider information in an isolated manner or direct their attention idiosyncratically from one source to others without connecting them.

In studies of reading comprehension, a popular approach is to capture text content through analyses of text structure, an important aspect of expository text as discussed earlier. To do so, previous research has used concept maps (Kinchin et al., 2000; Kintsch & van Dijk, 1978), the landscape model (van den Broek et al., 1999), and knowledge structure (Clariana & Wallace, 2007; Li & Clariana, 2019). These methods are aimed at describing not just the text characteristics, but the reader’s or learner’s mental representation of acquired knowledge after reading. A concept map is a simple visualization to sketch out information around a topic. It reflects how readers integrate text information into their existing understanding of a topic. Different from concept maps, the landscape model is a computational method to model how the text elements (which are likely to be conceptually linked together) are activated, along with modeling the reader’s attention and working memory as reading unfolds. Fluctuation of these activations along reading gradually evolves into a landscape, which reflects a reader’s mental representation of a given text. Finally, knowledge structure uses analyses that resemble the network science approach, albeit in a simpler form, in that they can be visualized in the form of a network with nodes (representing concepts) linked through edges (indicating relationships). Knowledge structure can reflect the interaction between a reader and a given text: during the reading process, knowledge components are transmitted from the author to the reader, and as a result, the reader eventually develops a mental representation of the newly acquired knowledge referred to as knowledge structure (Clariana, 2010; Jonassen et al., 1993).

Concept maps, landscape models, and knowledge structures can be mathematically described through network metrics. Hence, application of network analytic approaches such as those piloted by Zhu and Feng (2015) can advance eye-tracking research in reading by providing a context-based visualization of the reader’s attention allocation during information intake. By transforming visual scanpaths into a network where fixations are represented by nodes and saccades by edges, such a graph representation can lead to an increased understanding of individual differences along the process of reading. Quantification for comparing the different visualizations can then be enabled by detailed graph metrics.

Several network metrics have been considered in previous language-related literature. These metrics are designed to provide information about the structural properties of the network being examined. Zhu and Feng (2015) specifically suggested metrics like density, centrality, and clustering measures to be used when it comes to analyzing eye-tracking data. Below we provide a brief description of a few metrics that have been widely used in network science research and that are relevant to the analyses of eye-movement data (see discussion below and under Method).

### Network metrics

#### Density

**Fig. 2:**
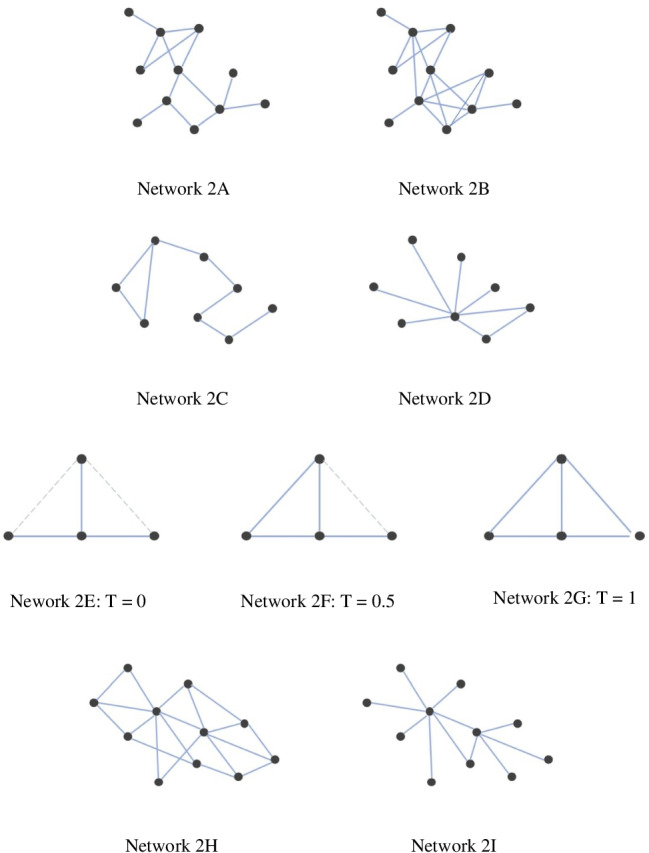
Illustrations of network types: 2A & 2B (density); 2C & 2D (centrality); 2E, 2F & 2G (transitivity); 2H & 2I (small-worldness)

Graph density represents the proportion of actually connected edges among all possible connections (links that could potentially exist between two random nodes). For instance, network 2B in Fig. 2 would have a higher density score compared to network 2A. The range of graph density goes from 0 to 1, with 0 being the least dense, and 1 being the densest network (Coleman & Moré, 1983). In a densely connected graph, the number of edges will be close to the maximal possible number of edges. Density is one of the most widely used network metrics as a holistic measurement of transactions among nodes in a network. When it comes to eye movement data, if a reader’s network is high in density, it suggests that a great deal of fixations and saccades are made going through a text with a small number of skips.

#### Centrality

Freeman’s calculation of maximum betweenness centrality (Freeman, 1978) provides a global measurement of how much variation there is in the node degree among all nodes in a network, where node degree captures the relative importance of a given node. More specifically, the degree of node *i* can be calculated as *C*_*D*_(*i*) *= deg* (*i*), with *deg* counting for the number of edges attached to node *i.* The value of graph centrality according to Freeman’s calculation ranges from 0 to 1, with 0 referring to a chain-like structure, and 1 referring to a spoke-like structure.

**Fig. 3:**
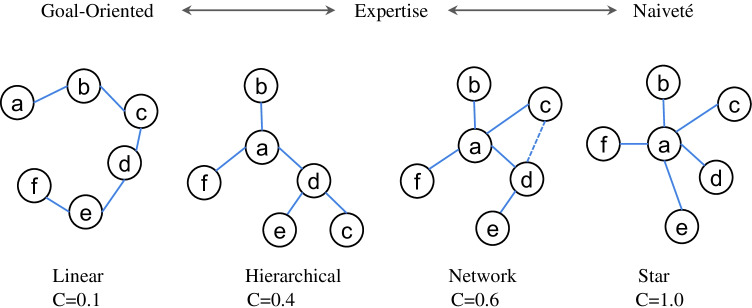
Graph centrality of different concept maps. Low centrality shows goal-oriented learners’ linear organization; medium centrality indicates expert learners’ hierarchical organization; high centrality represents naiveté learners’ star-like organization (Clariana et al., 2011)

Freeman’s graph centrality metric can reflect structural characteristics of the input material, at least for small networks. It is also considered as one of the most basic and efficient metrics to reflect a network’s form. Previous studies focusing on network centrality have demonstrated that as one node increasingly develops direct connections with other nodes in a network, this node will become more central in the network (i.e., the principle of preferential attachment) with a concomitant increase of the global centrality score as well (Clariana et al., 2013; Mak & Twitchell, 2020). Network 2D as shown in Fig. 2, for example, exhibits higher centrality compared to network 2C. Clariana et al. (2011) defined a conceptual topology using Freeman’s graph centrality to characterize network structures, with 0–0.2 representing a “linear” form, 0.2–0.4 a “hierarchical” form, 0.4–0.6 a “network” form, and 0.6–1 a “star” form (see Fig. 3 for an illustration of the topology). The centrality metric can potentially reflect a reader’s tendency to either focus more on the central topic or distribute relatively equal attention to all contents. Therefore, the centrality metric can provide insights on how readers handle repetitive words and function words that could serve as potential “hubs” in a network. This metric is also sensitive to the structure of the text itself—if we simply sew up all words in a text sequentially into a network, the network of an expository text will usually be more centralized than one of a narrative text. This is because scientific texts usually aim to illustrate a specific topic by drawing relative descriptions, whereas narrative texts generally follow a timeline that leads to a more linear structure (Clariana et al., 2014). Thus, the centrality metric is a good example of applying network metrics to capture the structure of a text.

#### Transitivity

Transitivity (also known as global clustering coefficient) is essentially a measure of triplet structure counting for the fraction of all possible triangles contained in a network. It represents the likelihood for two nodes to be connected if they share a mutual neighbor (Newman & Park, 2003). Its value *T* goes from 0 to 1 as shown in Fig. 2 networks 2E–2G : A network (2G) with *T* = 1 has all possible edges with the given nodes. The transitivity metric can be specifically useful to study regressive fixations in eye movements—if a reader’s network possesses high transitivity value, then there is likely a large amount of regression going on along the reading process. This is because the transitivity metric essentially measures the number of closed triangles in a network, which would be formed by regressive saccades.

#### Global efficiency

Global efficiency measures the average inverse shortest path lengths (i.e., the number of edges linking two different nodes in a shortest path) among all possible pairs of different nodes in a network (Ek et al., 2015; Latora & Marchiori, 2001). It is typically used as a measure of the capacity for parallel information transferring in a network, as this measure is estimated based on all pairs of nodes in the network. A network with high global efficiency is supposed to be more efficient as information exchange can happen simultaneously and quickly on multiple paths. The value of global efficiency goes from 0 to 1: A network with a value of 0 indicates that no edge exists between nodes and it is a disconnected network; a network with a value of 1 indicates that there is an edge between every pair of nodes in this network and it has the maximum global efficiency (Ek et al., 2015; Latora & Marchiori, 2001). Networks derived from visual scanpaths that contain repetitive fixations on various words should exhibit a higher global efficiency score, since repetitive fixations can lead to “hub” nodes (i.e., the important central terms) that provide alternative shortcuts among nodes. Therefore, the global efficiency metric can be highly relevant to reading comprehension, given previous studies suggesting the number of fixations, skips, and regressive fixations to be tightly linked with the reader’s comprehension ability.

#### Small-worldness

Small-world networks are expected to exhibit both high segregation and integration (Rubinov & Sporns, 2010). They are defined as highly clustered networks with approximately the same path length of an equivalent random network (Watts & Strogatz, 1998). Measurement of small-worldness takes both clustering coefficient and average path length into account. As illustrated in Fig. 2, network 2I possesses a small-world property whereas network 2H does not. Humphries and Gurney (2008) established a precise measurement of small-worldness that evaluates clustering and path length simultaneously. According to their quantitative definition, a network with an S value >1 can be counted as a small-world network. Such a network possesses more interconnected clusters and shorter averaged path lengths compared to a matched random network. The S value can range from 0 to over 1000. The real-world internet system, for instance, has an S value of 1093. Gao et al. (2014) compared six different languages including Arabic, Chinese, English, French, Russian, and Spanish to see the differences and similarities shared in human language. Interestingly, the co-occurrence networks of all languages being examined exhibited small-world properties. With respect to reading comprehension, the small-worldness metric can capture whether readers tend to land their fixations on reoccurring (repetitive) function words—the number of fixations on these function words (as opposed to less repetitive content words) suggests the degree of balance between a network’s global and local connections in a visual scanpath network.

How the above network metrics can be leveraged for the understanding of reading comprehension based on visual scanpaths will be examined in the current study, as we discuss below.

### The current study

Given the network metrics discussed above, the application of quantitative network analyses on eye-movement data from text reading becomes possible and useful. Different network metrics reflect different higher-order structure and characteristics of a given network, and may reveal important information processing strategies during the reading of texts. As has been piloted by Zhu and Feng (2015) using a simple between-group network comparison approach to examine math problem-solving ability, network metrics can also be applied to investigate individual differences during reading comprehension, especially in examining differences between skilled readers and less skilled readers.

Previous studies have not applied network-based analyses on readers’ eye-movement data obtained from text reading. Our study is aimed at understanding readers’ information processing in the context of both the structure of the reading material and characteristics of the reader, and providing an intuitive visualization of the process during which the reader constructs mental representations of the knowledge acquired from reading. In terms of text material, we targeted expository text in the context of scientific text reading. Eason et al. (2012), among others (see van den Broek, 2010 for a review), have suggested that reading comprehension can significantly vary along with text type. As discussed earlier, expository text imposes a higher demand on readers to organize and integrate target information in a structured fashion.

The current study applies a network science approach to a set of data collected from students’ reading of expository science text (see Method for details of the data). In the first step, each reader’s word-level scanpaths during reading were extracted and aggregated into an individual knowledge scanpath network. In constructing the scanpath network, we treated words that received fixations as nodes, and between-fixation saccades as edges. In the second step, metrics based on the visual scanpath data were computed as quantitative descriptions of the network, which include density, centrality, transitivity, global efficiency, and small-worldness. Through these two steps, we aimed to address the following questions: (1) Could scanpath networks serve as an informative and reliable representation of the reader’s comprehension process? (2) What can scanpath networks tell us about the individual differences in reading comprehension between skilled readers and less skilled readers?

With respect to the first question, we hypothesize that the visual scanpath network will represent the reader’s comprehension ability, with metrics including density, centrality, global efficiency, transitivity, and small-worldness significantly correlated with participants’ reading comprehension outcomes. The second question asks if the network metrics may reveal individual differences during the reading process. Given that previous studies (e.g., Ashby et al., 2005; Chace et al., 2005) have indicated that skilled readers exhibited fewer fixations, more skips, and fewer regressions during reading, we hypothesize that the scanpath networks derived from eye-movement data may show higher centrality, lower density, lower small-worldness, lower global efficiency, and lower transitivity for skilled readers, as compared with less skilled readers. These differences derived from network science measures will lead to significant insights into individual differences in reading comprehension of expository texts.

## Method

### Participants

Fifty-two participants (24 men) aged between 18 and 40 years (mean age ± SD = 22.85 ± 4.66 years) were recruited from Pennsylvania State University (PSU). Participants were all right-handed, native English speakers with normal or corrected-to-normal vision, and no past history of mental or neurological disorders. The study was approved by the PSU Institutional Review Board (IRB) and was performed in accordance with the ethical standards required by the IRB. Written informed consent was obtained from all participants before they took part in the study. 

### Material

The study material included five expository science texts designed by Follmer et al. (2018), introducing the scientific concepts of Mars exploration, permutation versus combination (math concepts), the electrical circuit, Global Positioning System (GPS), and supertanker (ecology). The five expository texts can be found in Appendix A. Each text contains 30 sentences and about 300 words on average, and all designed with a consistent wording style (see Appendix B for an overview of the psycholinguistic variables of texts).

### Procedure

An EyeLink 1000 Plus long-range eye tracker (SR Research, 2016) with a sampling rate of 1000 Hz was used to collect eye-movement data. Only the reader’s right eye was recorded for movements during reading. Before the reading task, a 13-point calibration and validation adjustment were conducted for tracking precision. Eye-movement data in the current study were collected in the magnetic resonance imaging (MRI) scanner through a fixation-related functional MRI (fMRI) method (Henderson et al., 2016; Richlan et al., 2014) so that naturalistic reading could be performed in the scanner. Reading in the fixation-related fMRI was done through the presentation of materials on a reflection mirror inside the MRI, which could exacerbate eye drifting caused by multiple lines in the reading material. In light of this issue, previous studies using fixation-related fMRI typically presented the reading material word by word or sentence by sentence, or in very short paragraphs (Carter et al., 2019; Henderson et al., 2016; Schuster et al., 2020). Because our expository texts were generally longer (around 300 words) than those used in previous studies, to control for potential eye drifting while providing a natural reading experience, we presented the text to the participants in a sentence-by-sentence rather than whole-paragraph format. Previous empirical studies have confirmed that reading outcomes are comparable between sentence-by-sentence and whole-paragraph text formats in expository scientific text reading (Follmer et al., 2018).

**Fig. 4:**
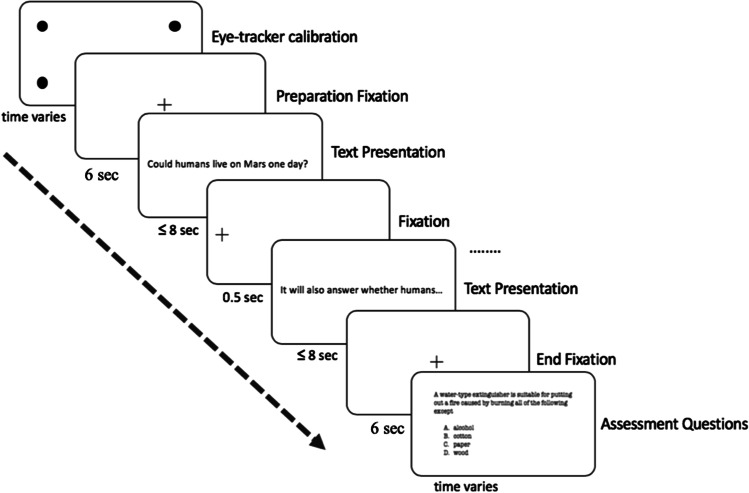
Data collection overview. The ≤ 8 s time duration for text presentation reflects the self-paced reading setting. Once text presentation is finished, participants responded to 10 multiple-choice assessment questions, one by one

E-Prime 2.0 (Schneider et al., 2002) was used to control the presentation of texts on the screen. Presenting screen size was 35.7 cm × 57.2 cm, and the average word length on the screen was 3.08 cm. A reader’s visual angle when fixating on a word was about 1.14°. Figure 4 presents a flowchart of the entire reading task procedure. The eye-tracking data were extracted from a larger reading brain project, in which participants’ behavioral, eye-movement, functional and structural brain imaging data were simultaneously collected in the same study (see the fMRI data presented in Hsu et al., 2019, and the *Methods* for details).

Before the appearance of each sentence, a fixation cross was shown on the left side of the screen. Participants were instructed to focus on the cross while anticipating the presence of sentences. Time interval between appearance of the fixation cross and the next sentence was 500 ms, except for the first fixation cross that lasted for 6000 ms for participants to get prepared. To enable a natural reading experience, we set the reading to be self-paced. When finished with one sentence, subjects could press a button on the response box to advance to the next sentence; otherwise, the screen would automatically advance to the next sentence in 8000 ms. After the self-paced reading task, 10 multiple-choice assessment questions related to the text content were presented to participants to evaluate their comprehension of the text. All participants took part in a practice session before performing the real experiment.

The Gray Silent Reading Test (GSRT) was used to examine participants’ reading competence (Wiederholt & Blalock, 2000). It is a standardized, age-based reading test (with varying levels of difficulty for readers of ages 7 to 25). The test materials contain 26 separate texts, with each text followed by five multiple-choice questions. Reading materials of the text are arranged into 13 levels based on difficulty. When a reader answers all five questions of a text correctly, a basal is triggered and all questions in the lower (easier) levels are automatically considered correct. When a reader answers three out of five questions incorrectly, a ceiling is established and all questions in the higher (harder) levels are automatically considered incorrect. In this case (accuracy lower than 40%), the test will stop and the final comprehension score will be generated. This developmentally sequenced reading test provides a standardized and reliable measurement of readers’ comprehension ability.

### Data analysis

In preparation for data analysis, participants’ reading comprehension ability was computed as a summed normalized score that includes their post-reading assessment questions, GSRT score, and total reading time. The total reading time refers to the average total time each participant spent reading a given text. All three scores were transferred into z-scores before the combination. This new combined score (which will be referred to as “comprehension ability score”) accounted for both participants’ reading performance (post-reading assessment score + GSRT score) and reading speed (reversed score of total reading time = max − raw reading time). Through this combined score, we expect that readers who read more efficiently and accurately would receive a higher comprehension ability score. Note that we did not treat the three scores (i.e., assessment accuracy, GSRT score, reading time) separately to measure reading comprehension, as we had no independent evidence to believe that each score would be correlated separately with the network metrics in our study. Further, the use of a combined score as an index of reading comprehension was based on significant evidence from the literature that neither reading accuracy nor reading speed alone could account for an individual’s reading comprehension ability (e.g., Hudson et al., 2008; O'Connor et al., 2010; Tijms, 2007).^1^

For eye-movement data collection, AOI was set to the word level. Due to fixation drifting caused by the declining accuracy of calibration over time, eye-movement data were readjusted as follows: for fixations shorter than 40 ms or longer than 1000 ms, they were excluded using the “clean” function in the Data Viewer™ software (Li et al., 2014; SR Research, 2016); for fixations falling outside (above or below) the range of predefined target regions where sentences were presented, manual adjustment was performed using the Data Viewer™ software (SR Research, 2016). Instead of using auto-adjustment which brings all fixations onto one horizontal line, we performed trial-by-trial correction only along the *y*-axis (vertical adjustment) so as to protect readers’ original eye fixation patterns. Within our participant group, less than 10% of the data needed to be manually corrected in this fashion.

Individual scanpaths were first extracted based on real-time word-level fixation onsets, reflecting the actual reading intake for each reader. Visual scanpaths do not easily render themselves to quantifiable comparisons across individual readers or texts. To quantify the visual scanpaths, we converted the scanpaths of each participant going through the entire text to a network, where each fixated word was encoded as a node and each saccade between fixations as an edge. Reoccurring words in the text were merged as one node. In other words, each graph was made up of all words that the participant had fixated on and all the transitions across these words. The weights of the edges indicated the number of saccades being made between two words and their directions indicated the saccade directions. The graphs were computed in the form of adjacency matrices (see Table 1), with rows and columns representing words that received fixations (i.e., where saccades started and ended), and cell values of each entry representing the number of transitions/saccades between two corresponding words, regardless of directions. In computing the actual networks, the directions of saccades were included in the edges so as to differentiate between forward saccades and backward regressions.

**Table 1:** Example of network matrix*

	Could	human	on	mars	day	scientist	this	question
could	⎻	1	0	0	0	0	0	0
human	0	⎻	1	1	0	1	0	0
on	0	0	⎻	1	0	0	0	0
mars	0	1	0	⎻	3	0	0	0
day	0	0	0	3	⎻	0	0	0
scientist	0	0	0	0	0	⎻	1	0
this	0	0	0	0	0	0	⎻	1
question	0	0	0	0	0	0	0	⎻

* Words in the rows and columns indicate the words that received fixations (example from subject #2). Cell values indicate the number of transitions/saccades between two corresponding words, regardless of directions.

To visualize the different reading behaviors of skilled and less skilled readers, we averaged the adjacency matrices of 10 participants with the highest comprehension ability scores and 10 with the lowest scores respectively, and visualized the resulting graphs with NetworkX (Hagberg et al., 2008). Moreover, in order to maximize informativeness and intuitiveness of these averaged graphs so as to focus on the most fixated terms, we applied a mask to the two networks filtering out nodes and edges with low representativeness: We only included nodes with degrees greater than five and edges with weights greater than 0.3. The degree of a node is defined as the sum of weights of all its connected edges. In the context of eye movements, it is equivalent to the total number of saccades that landed on and departed from a given word. This masking procedure effectively served to reduce noise in the data caused by network aggregation across multiple individuals.

To facilitate quantitative inference of individual comprehension outcome based on eye-movement patterns, we further carried out in-depth computations of graph metrics for each scanpath network. Selection of the specific metrics was inspired by work on network measures applied to cognitive studies (e.g., Rubinov & Sporns, 2010). Based on metric relevancy in the context of eye-movement patterns, the following metrics were included in our data analyses (see also a conceptual discussion of the metrics in the Introduction):

***Density*** measures the wiring cost of a network, which is defined as the sum of weights of the edges in the graph divided by the number of possible edges. For a directed weighted graph *G*=(*V*, *E*), where *V* denotes its nodes and *E* denotes its edges, its density is

$$D=\frac{\sum_{i\in N,j\in N,i\ne j}{w}_{ij}}{N\bullet \left(N-1\right)}$$

where *N* is the number of nodes and *w*_*ij*_ is the weight of the edge from node *i* to node *j*.

***Centrality,*** when measured at a single-node level, quantifies the number of times a node acts as a bridge along the shortest path between two other nodes. When measured at a global level, centrality reflects how important its most central node is in relation to all the other nodes in a network. Freeman’s maximal betweenness centrality is one of the well-established global centrality metrics that measures the variation of the node degree scores (*C*_*d*_(*i*) = deg(*i*)/(*n* − 1)) among nodes. Let *C*_*i*_ be the degree of the node *i* and *C*_*max*_ be the maximum degree of all nodes in a graph, the maximal betweenness centrality is calculated as:

$$C=\frac{\sum_{i\in N}\left({C}_{max}-{C}_i\right)}{\left(N-1\right)\left(N-2\right)}$$

***Transitivity*** measures graph segregation, which is defined as the relative number of triangles in the graph divided by total number of connected triples of nodes. In the current study, computation of this metric was implemented as:

$$T=\frac{\sum_{i\in N}{t}_i}{\sum_{i\in N}\left[{k}_i\left({k}_i-1\right)-2\sum_{j\in N}{w}_{ij}{w}_{ji}\right]}$$

where *k*_*i*_ is the degree of the node i (i.e., sum of weights of its edges) and *t*_*i*_ is the number of directed triangles around the node i: ${t}_i=\frac{1}{2}\sum_{j,h\in N}\left({w}_{ij}+{w}_{ji}\right)\left({w}_{ih}+{w}_{hi}\right)\left({w}_{jh}+{w}_{hj}\right)$.

***Global efficiency*** measures graph integration, which is the sum of inverse shortest distance between every possible pair of nodes, with the distance of two connected nodes defined as the inverse of the edge weight. The global efficiency of an individual network was computed as:

$$E=\frac{1}{N}\sum_{i\in N}\frac{\sum_{j\in N,j\ne i}{\left({d}_{ij}\right)}^{-1}}{N-1}$$

where *d*_*ij*_ denotes the shortest distance between node *i* and node *j*.

***Small-worldness*** measures both segregation and integration. A small-world network is both highly segregated and integrated. It can be computed as a clustering coefficient of the graph normalized by that of a randomized graph with the same number of nodes and edges divided by its characteristic path length normalized by that of the randomized graph. In short, small-worldness in the current study was calculated as

$$S=\frac{C/L}{C_{rand}/{L}_{rand}}$$

where the clustering coefficient of the network is $C=\frac{1}{n}\sum_{i\in N}\frac{2{t}_i}{k_i\left({k}_i-1\right)}$ and the characteristic path length of the network is $L=\frac{1}{n}\sum_{i\in N}\frac{\sum_{j\in N,j\ne i}{d}_{ij}^{-1}}{n-1}$.

## Results

Prior to data analysis, a summary of descriptive statistics based on the network metrics as described above is presented in Table 2. Metrics including density, centrality, transitivity, global efficiency, and small-worldness were calculated for each scanpath network. Subsequently, each metric obtained from the five texts was averaged for each participant. Finally, average metrics were used for the relevant statistical analyses as reported below. To answer the first research question of whether visual scanpath can be used as a valid indicator of readers’ comprehension outcomes, we focused our analyses on the relationship between readers’ comprehension ability score and their scanpath network metrics. Pearson correlation coefficients were calculated among all related variables (see Table 3). The correlation analysis showed several significant associations between an individual’s reading comprehension outcome and their scanpath network metrics of density (*r*(50) = −.39, *p* < .01), centrality (*r*(50) = .45, *p* < .001), small-worldness (*r*(50) = −.12, *p* = .41), global efficiency (*r*(50) = −.33, *p* < .05), and transitivity (*r*(50) = −.51, *p* < .001). These correlations indicated that the scanpath networks reflect different degrees of skillfulness in reading: the more skilled the readers were, the more efficiently they read, and the higher/lower the corresponding network metrics became (with the exception of small-worldness; see discussion below).

**Table 2:** Descriptive statistics of the network metrics and related variables

	Minimum	Maximum	Mean	Std. deviation
Density	.019	.030	.024	.002
Small-worldness	1.300	1.890	1.590	.137
Centrality	.0026	.0048	.0035	.0004
Transitivity	.039	.108	.074	.016
Global efficiency	.324	.374	.345	.011
Comprehension ability score	−2.647	3.471	1.088	1.472

*N* = 52. Unit of readers’ total reading time is measured in milliseconds.

**Table 3:** Correlation among related network metrics of reading

	Density	Small-worldness	Centrality	Transitivity	Global efficiency
CA Score	−.392**	−.116	.446**	−.505**	−.329**
	.004	.414	.001	.000	.017
Density	-	.426**	−.453**	.829**	.849**
	-	.002	.001	.000	.000
Small-worldness		-	−.208	.516**	.383**
		-	.139	.000	.005
Centrality			-	−.628**	−.384**
			-	.000	.005
Transitivity				-	.753**
				-	.000
Global efficiency					-
					-

*N* = 52. CA Score stands for comprehension ability score.

** Correlation is significant at the 0.01 level (two-tailed). * Correlation is significant at the 0.05 level (two-tailed).

To further examine individual differences, a between-group comparison was performed, with participants divided into skilled readers (top half 26 readers) and less skilled readers (bottom half 26 readers) based on their comprehension ability score. An independent samples *t*-test showed that skilled readers and less skilled readers’ average scanpath networks differed significantly in density (M_skilled_ = .024, M_less-skilled_ = .025, *t* (50) = −2.73, *p* < .01, Cohen’s *d* = .76), transitivity (M_skilled_ = .067, M_less-skilled_ = .081, *t*(50) = −3.56, *p* < .001, Cohen’s *d* = .99), global efficiency (M_skilled_ = .341, M_less-skilled_ = .349, *t*(50) = −2.66, *p* < .05, Cohen’s *d* = .74), small-worldness (M_skilled_ = 1.544, M_less-skilled_ = 1.635, *t*(50) = −2.51, *p* < .05, Cohen’s *d* = .70), and centrality (M_skilled_ = .004, M_less-skilled_ = .003, *t*(50) = 2.53, *p* < .05, Cohen’s *d* = .70).

**Fig. 5:**
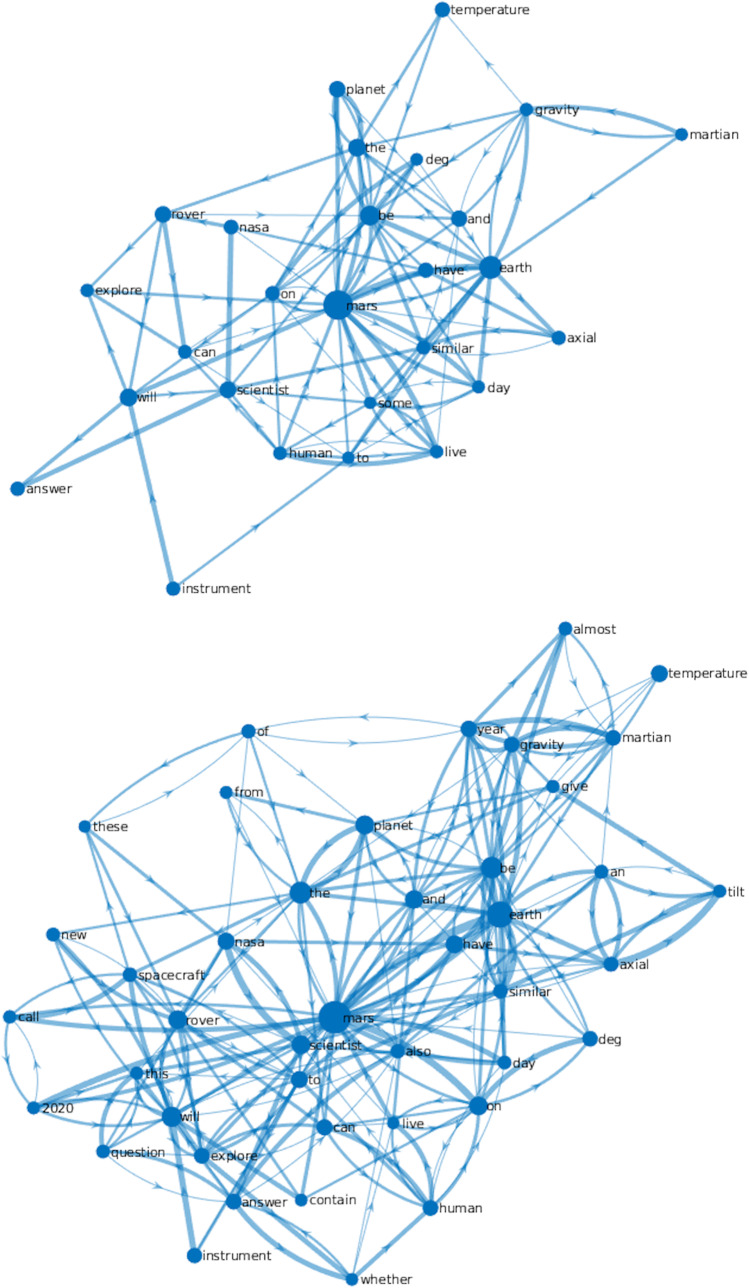
Averaged scanpath networks of the Mars Exploration text for skilled readers (top) and less skilled readers (bottom). Nodes represent the words that received fixations (mask applied, repetition merged to enable readability), and edges represent weighted and directed saccade transitions (including forward saccades and regressions).

These overall significant between-group differences led us to further visually examine the scanpath networks of the Mars Exploration text for highly skilled readers (top 10 performers of the participants) and much less skilled readers (lowest 10 performers of the participants) ranked by comprehension ability score. Figure 5 provides a clear visualization of the differences, with larger nodes indicating higher values of node degree/density, and thicker edges more transitions/saccades between nodes. The two topic words in the text—“Mars” and “Earth” —serve as hubs in both networks. As larger nodes represent the keywords of the text, it is natural that they would receive more repetitive fixations in the less skilled readers’ network compared with those in the more skilled readers. Less skilled readers also spent more time on functional words (41.46%) as compared with more skilled readers (29.63%).

## Discussion

### What scanpath networks reveal about reading comprehension and individual difference

The current study has been designed to examine the link between readers’ comprehension of expository scientific text and their eye-movement patterns during reading by using the analytic method from network science. To achieve this goal, we asked two research questions: (a) Would scanpath networks serve as a useful and reliable indicator of a reader’s comprehension process? (b) What can scanpath networks tell us about the processing differences in reading comprehension between skilled readers and less skilled readers? To answer these questions, we introduced network science metrics and analyses as they are applied to eye-movement patterns during text reading. Specifically, we transformed raw eye-tracking data first from word-level fixation onset times to visual scanpaths. We then used the scanpaths to derive networks and calculated the relevant network metrics. This approach enabled us to identify and visualize the relationship between readers’ eye-gaze patterns during reading and their comprehension outcomes. By utilizing network analysis, individual readers’ scanpaths could be quantified and further compared, regardless of the number of fixations that readers make in reading the same text.^2^ This enabled us to investigate the process of readers’ information encoding in a less rigid manner, without setting pre-defining keywords (as is typically done in other methods; see 1.3) that could constrain the variance among participants.

First, individual-level network analyses confirmed our hypothesis that network metrics are significantly correlated with reading comprehension through a combined reading score in our study. The graph metrics of centrality showed a positive correlation, whereas density, global efficiency, and transitivity showed negative correlations with readers’ comprehension ability. These results supported our assumption that scanpath networks constructed from visual scanpaths can capture the reader’s comprehension of scientific texts. Second, group-level network analyses between skilled and less skilled readers also supported our hypothesis that the scanpath network method can be a valid tool for investigating individual differences in reading comprehension. Our findings indicated that skilled readers’ scanpath networks exhibited higher centrality, lower density, lower global efficiency, and lower transitivity. These patterns are consistent with previous eye-tracking studies suggesting that skilled readers make fewer fixations, more skips, and fewer regressions during reading. For example, in previous eye-tracking studies, word skipping happens more often on short-length, high-frequency, and predictable words (Engbert et al., 2005; Kliegl et al., 2004; Rayner et al., 2011; Reichle et al., 1998). If a skilled reader goes through a text skipping many predictable, repetitive, and short-length function words with little checking on previously visited words, then the network constructed by this reader’s visual scanpath, as we have presented here, would be less dense as a result of fewer fixations and fewer overall transitions (both advancing and regressing).

As mentioned earlier, because a text has a specific structure, the centrality metric will be greatly influenced by the text structure itself. This is especially true given the way reading material was presented in the current study: Since participants read in a sentence-by-sentence manner, their visual scanpaths were first constructed on a sentence level, and then connected by fixations on repetitively appearing topic words and function words such as “Mars,” “Earth,” “supertanker,” “the,” and so on. In this case, the overall structure of readers’ scanpath networks was to some extent constrained by the text structure and the way text was presented. What differentiated the skilled readers from less skilled readers could be the manner in which their scanpath networks branched out upon having only partially defined structure: skilled readers’ networks exhibited more sparsely clustered branches as a result of more focused scanpaths with fewer function words, short-length words, and irrelevant words. In contrast, less skilled readers’ networks grew out denser connected branches that diverged from the hub nodes, which led to a lower centrality value. Such topological structures reveal the different reading processes and reflect individual differences with a clear overall visualization.

Such tendency during the reading process can also lead the transitivity metric to become higher in less skilled readers’ networks. The transitivity metric essentially measures the number of triplet nodes in a network. Along the reading process, regressive fixations on previously visited words can increase the number of closed triplets in one’s scanpath network. Repetitive fixations on function words and reoccurring topic words can also create more triplets in the network. Therefore, it is not surprising that the transitivity metric exhibited a negative correlation with readers’ comprehension in our study.

As for the global efficiency metric, it is an inverse measurement of the shortest path length (the shorter, the more efficient), reflecting how efficiently a network exchanges information. This property ensures optimal information exchange within a network given limited space and resources. Broadly speaking, it measures how “cost-efficient” a given structure is. Many real-world structures such as subway systems, airline networks, and even neural networks in our brain exhibit high global efficiency properties (Bullmore & Sporns, 2009; Guimera et al., 2005; Latora & Marchiori, 2002; Li & Grant, 2016). On a global scale, the shorter the path length (number of edges) between any two random nodes, the higher a network’s global efficiency score. Since skilled readers tend to skip more words and make fewer transitions between words going through a sentence, fewer edges would be built among nodes in their scanpath networks. If a skilled reader is more likely to extract information from repetitive and short-length words (e.g., function words) through parafoveal preview without having to land an actual fixation on them (Blanchard et al., 1989; Wang et al., 2014), then there should be fewer surrounding nodes being directly connected to hub nodes in such a reader’s scanpath network. Given such a sparsely connected network, the average shortest path length between any two random nodes would be longer, therefore leading to a lower global efficiency score.^3^

Compared to other metrics, small-worldness is a more complex measurement since it captures both segregation and integration of a network. Humphries and Gurney (2008) precisely described how small-worldness changes as a function of network topology: as a measure of network clustering controlled for path length, it exhibits a U shape and peaks at the point where the trade-off between global clustering coefficient and path length reaches maximum (for more details see Figure 1 in Humphries & Gurney, 2008). In other words, a network with small-world phenomenon should have local clusters around sub-hubs (high clustering coefficient), with these hubs then making global connections allowing for an efficient overall connection within the network (shortest path length), instead of having a large number of global connections that will be resource costly. Such property (i.e., a non-monotonic curve) explains why the small-world metric did not show a significant linear correlation with readers’ comprehension ability in this study. This could be caused by skilled readers’ tendency to skip not only function words, but also those repetitive topic words when they occur frequently and are short in length (e.g., “Mars,” “GPS”). After repetitive exposure to these topic words, the skilled readers are able to extract the semantic information without having to land a fixation on them. As a result, the number of sub-hubs and the amount of edges linking back to central hubs both decreased, canceling out the effect that could potentially be revealed by the small-worldness metric.

Results of the current study made it clear that through quantification of scanpath networks, graph metrics can serve as a useful and reliable index of a reader’s comprehension ability. By applying network science to eye-movement data, we were able to transform readers’ information intake process into intuitive visualizations that also reveal underlying processing differences between skilled readers and less skilled readers. This contrasts with previous scanpath studies that were mostly focused on qualitative patterns of how readers orchestrated sequential fixations. For example, von der Malsburg and Vasishth (2011) proposed an algorithm to estimate scanpath similarity using eye movements and divided the reading patterns into different categories. This algorithm has been applied to reading patterns from readers at different levels (e.g., fluent, intermediate, and beginner) (see also Parshina et al., 2021). However, this approach regards each fixation on each word as an individual unit, ignoring the crucial relationships among words during reading comprehension. By contrast, the scanpath network in the current study counted the same word as a node and employed direction and strength of edges to collectively reflect connections among words. The latter approach is therefore more likely to capture the overall structure at a global rather than an individual word level. One of the key tasks for the reader is to build associations in structured or hierarchical relations among words (Li & Clariana, 2019), and the scanpath networks in the current study capture the overall structure of the reading process in terms of how words are connected by the reader as reading unfolds. Our approach provides a new complementary method for eye movement studies of reading comprehension.

### Limitations and future directions

One potential limitation with our current study is that our participants read the text in a sentence-by-sentence manner, which is better than word-by-word reading but is still different from the naturalistic whole-paragraph reading experience. However, in a recent paper Follmer et al. (2018) showed that regardless of whether the same text was read in a sentence-by-sentence manner or paragraph-by-paragraph manner, the underlying knowledge structure derived (as assessed by Multidimensional Scaling or MDS) is actually similar. Of course, if whole paragraphs were presented to participants, readers would be given the flexibility to check across sentences, and a more robust connection between the network metrics and comprehension ability might have surfaced. Specifically, if forward and backward saccades are allowed to happen across sentences, connections among nodes (words) may include edges (saccades) both within and across sentences. In the current study, all the network metrics have been partially constrained by the format of text presentation, as earlier discussed.^4^ This constraint, on the other hand, could help reveal interesting patterns between skilled versus less skilled readers. For example, because readers cannot regress back across sentences, skilled readers may be able to hold information across sentence boundaries, due to their higher working memory (see Li & Clariana, 2019 for a review of text reading and working memory). Therefore, skilled and less skilled readers would show different centrality and global efficiency scores. The relationship between working memory and executive control and network metrics for reading should be further pursued in future studies.

Previous eye-tracking studies have investigated reading comprehension mainly based on word-level or sentence-level eye-movement features. The current study attempted to link eye movements with the text content. Compared with traditional measurements focusing on eye-movement properties like averaged fixation duration, skip rate, overall fixation count, and so on, the scanpath network approach allows us to bring in more precise information regarding each reader’s actual information processing, and reveal the overall underlying structure of eye movements dependent on text properties. The network approach adopted in the current study would therefore also have practical utility for researchers who would like to get a glance of the overall structure through visualization. In future studies, it would be important to develop visualization tools to automatically conduct such analyses on eye-movement data to enable this inquiry.

## Resources and tools

The network science approach to the study of reading comprehension is a relatively new approach, but we have demonstrated here the utility and power of this approach to the understanding of reading patterns and individual differences in the context of comprehension of expository scientific text. Below we provide a few pointers to resources and tools that researchers can use to apply the methods presented in this paper.

To calculate scanpath networks from eye-movement patterns (see sections 1.3 and 2.4), one can use the EyeLink® Data Viewer Software from SR Research (the company that produces the EyeLink 1000 Plus eye tracker), in combination with data cleaning tools like SQL, Python, and Microsoft Excel. To calculate the network metrics used in this paper (see 2.4), one can use network analysis packages like NetworkX (Hagberg et al., 2008)—a collection of network manipulation and metric generation algorithms in Python. A variety of other network metrics and manipulation tools including boundary, d-separation, and dominance are also available in this package. Detailed source code and references can be found in Software for Complex Networks (2020).

Following the recent call of *Behavior Research Methods* on open access of data for research reliability and validity (Brysbaert et al., 2021), all data used in this article have been organized and uploaded onto the *OpenNeuro* platform under the dataset named “The Reading Brain Project L1 Adults” (see https://openneuro.org/datasets/ds002247/versions/1.0.0). We also provided very detailed documentation for the data acquisition and processing steps (see section 6 of the Reading Brain Project: Methods for Data Collection (L1 Adults); https://blclab.org/wp-content/uploads/2019/08/Reading_Brain_Methods_L1Adults.pdf), along with related information of behavioral and neuroimaging data collection and processing (sections 5 and 7), participant background and experiences (section 8), and appendices that contain instructions for the experiments and descriptions of the eye-tracking data file (section 10). Readers interested in applying network analyses on eye movement data (or related behavioral and neuroimaging data) can download our dataset and the documentation either from the *OpenNeuro* website mentioned above or from https://blclab.org/reading_brain/.

Finally, for general applications of the network science approach in cognition and language studies, one can consult a recent volume edited by Vitevitch (2019), and recent reviews by Karuza et al. (2016), Bassett and Sporns (2017), and Zaharchuk and Karuza (2021).

## Appendix A: Reading material

***Mars exploration***

Could humans live on Mars some day? Scientists ask this question because Earth and Mars are similar. Similar to Earth’s day, Mars’s day is about 24 hours long. Also, both planets are near the Sun in our solar system. Earth is the third planet and Mars the fourth planet from the Sun. Mars also has an axial tilt similar to Earth's axial tilt. An axial tilt gives both planets seasons with temperature changes. Just like Earth, Mars has cold winters and warmer summers. Like Earth, Mars has winds, weather, dust storms, and volcanoes. But in some ways, Earth and Mars are different. Differences include temperature, length of a year, and gravity. The average temperature is −81 °F on Mars, but 57 °F on Earth. A Martian year is almost twice as long as an Earth year. Earth’s gravity is almost three times stronger than Martian gravity. Given the similarities, can humans go to Mars and live there? NASA scientists want to answer this question. NASA oversees U.S. research on space exploration. NASA scientists send devices called spacecraft to explore Mars. The spacecraft carry rovers that can rove or move around. These wheeled rovers can explore characteristics of the planet. They can take pictures of mountains, plains, and dust storms on Mars. One of these NASA rovers is named Curiosity. Curiosity found evidence that soil on Mars contains 2% water. NASA has planned a new mission called Mars 2020. This mission will use a new car-sized rover to examine Mars. The new rover will contain additional instruments to study Mars. For example, one instrument will take images beneath Mars’s surface. Another instrument will attempt to make oxygen from carbon dioxide. Mars 2020 will help scientists answer important questions. It will explore whether there has been life on Mars. It will also answer whether humans can live on Mars in the future.

***Electrical circuit***

Engineers design electrical circuits to power homes and buildings. Electrical circuits are closed paths that electricity flows through. This means that the entire path must be connected like a loop. Imagine a simple circuit made with a battery and a piece of wire. Now connect each end of the wire to different ends of the battery. Your piece of wire and battery now make a circle or circular path. One end of the battery is positive and the other end negative. Electrons will flow across this circuit through the wire. You have just created a simple electrical circuit. Removing the wire from the battery will stop the flow of electrons. Now imagine that you want to connect the battery to a lightbulb. First the wire is cut in the middle and breaks the circuit. Then a lightbulb is inserted to reconnect the circuit. The electrical circuit is complete again and electrons are flowing. Electricity now flows through the lightbulb, lighting it up. If the circuit is now broken, the lightbulb will go out. You can also add a switch in the circuit to break it or connect it. This allows you to turn on or turn off the lightbulb. A simple electrical circuit contains several parts. These include a source, a path, and a resistor. The source provides the energy to an electrical circuit. In our example above, the source is the battery. Batteries can have different electric potential called voltage. The path is the closed loop with wires that connect to the source. Electrons flow through the path as electric currents. Electric currents increase with increased voltage. The resistor is any device that reduces the electric current. It creates resistance or impedance in the electrical circuit. In our example above, the resistor is the lightbulb. Electric currents decrease with increased resistance.

***Permutation vs. combination***

Two important math concepts are combinations and permutations. Combinations and permutations are similar in several ways. Both refer to sets of objects that you pick and arrange in order. A set could be the numbers 1 through 9, or available pizza toppings. For combinations and permutations, items are selected from a set. For example, we can select 1–3 from the set 1–9 to count and order. However, combinations and permutations are calculated differently. When we compute a combination, the item order does not matter. A combination of 1, 2, 3 is the same as the combination of 3, 2, 1. Another example of a combination is picking pizza toppings. Suppose you pick three toppings: cheese, pepperoni, and sausage. You can also say that you pick sausage, pepperoni, and cheese. Here you have a combination of three things in two different orders. But the pizzas you get are the same because the order doesn’t matter. However, order does matter when we compute a permutation. 1,2,3 and 3,2,1 are different permutations of these three numbers. In permutations, different orders of items have different meanings. Imagine you can pick only one for lunch: pizza, pasta, or rice. You say pizza is your favorite and rice is your least favorite. So your order of preference is (1) pizza, (2) pasta, and (3) rice. If pizza runs out, the waiter will give you pasta and not rice. But your friend may have a different order of preference. Your friend’s order could be (1) rice, (2) pasta, and (3) pizza. So two orders of the same items mean something quite different. Permutation allows us to count all the possible orders of items. Given three numbers 1,2,3, we can derive six permutations. These include 1,2,3 / 1,3,2 / 2,1,3 / 2,3,1 / 3,1,2 / 3,2,1. These sequences in different orders are different permutations.

***Supertanker***

How can engineers help prevent spills of oil from supertankers? Supertankers are huge ships that carry oil over the oceans. A supertanker can contain about a half-million tons of oil. A supertanker is the size of about five football fields. A supertanker’s cargo area could hold the Empire State Building. Most of the world’s oil is transported by these supertankers. Disasters occur when wrecked supertankers spill oil into the ocean. As a result of these oil spills, the environment is damaged. In 1967, a supertanker crashed near the shores of England. This crash resulted in washing ashore 200,000 dead seabirds. In 1989, another supertanker spilled oil into Alaska’s coast. The spilled 11 million gallons of oil caused 1000 otters to die. Oil spills from supertankers also kill drifting microscopic plants. These plants provide food for sea life, such as whales and shrimp. The plants also produce 70 percent of the world's oxygen supply. Oil spills result partly from limitations in engineering. Supertankers lack double bottom hulls for extra protection. Supertankers lack extra power and steering equipment for safety. Supertankers also have only one boiler to provide the ship power. Supertankers have only one propeller to steer the huge ship. Lack of such backup components causes problems during emergencies. Emergencies for supertankers are ocean storms and coastal reefs. Solutions to problems with supertankers include three tactics. Supertankers must be built with added hulls, boilers, and propellers. These provide extra safety, control, and backup in emergencies. Also, officers need top training to run and maneuver their ships. Supertanker simulators at some facilities provide top training. Finally, ground control stations should be installed near the shore. Ground control stations would act like airplane control towers. They would guide supertankers safely on the oceans along coasts. This ensures safety in shipping lanes and along dangerous coasts.

***GPS***

Global Positioning System (GPS) is a system that helps us navigate. GPS is a system of 24 or more satellites in space. These satellites orbit at about 12,000 miles above the Earth. Different satellites orbit the Earth in different locations. They circle the Earth along one of six orbits continuously. They send information to a GPS receiver on Earth. The information is transmitted across space via radio signals. Radio signals travel through space like sound waves through a canyon. Imagine you and your friend are at different sides of a canyon. You shout to your friend and she hears you after a short delay. This delay is the time that sound waves need to reach the other side. Similarly, the satellite in space sends a radio signal at one time. After a delay the radio signal arrives at the GPS receiver on Earth. The receiver records the precise time when the radio signal arrives. It then calculates the difference between these two times. This time difference is the travel time of the radio signal. The GPS uses this travel time to figure out how far the satellite is. It uses the formula “distance = time (T) × rate of transmission (C).” T is the travel time of the radio signal. C is the speed of light, more than 186,000 miles per second. T × C calculates the distance between the receiver and each satellite. Different satellites have different distances from the receiver. This information helps the receiver determine its location on Earth. The precise location is calculated based on geometry of distances. A GPS device that you carry in your car is a small receiver. Radio signals from the satellites are updated as the device moves. At least four satellites are involved to pinpoint the device’s location. GPS devices provide maps and directions that help people travel.

## Appendix B

**Table 4:** Statistics and psycholinguistic variables of texts*

Text metrics	Math	GPS	Mars	Circuit	Supertanker
**No. words**	308	307	310	304	302
**No. characters (excluding spaces)**	1491	1476	1528	1498	1676
**No. characters (including spaces)**	1798	1783	1838	1800	1978
**No. paragraphs**	1	1	1	1	1
**No. sentences**	28	28	31	30	31
**Avg. words per sentence**	10.7	11.3	10.3	10.0	9.7
**Avg. characters per word**	4.7	4.6	4.7	4.8	5.4
**Avg. characters per sentence (excluding spaces)**	53.25	52.71	49.29	49.93	54.06
**Avg. characters per sentence (including spaces)**	64.21	63.68	59.29	60.00	63.81
**Passive sentences %**	7.0%	10.0%	3.0%	10.0%	12.0%
**Flesch reading ease**	63.92%	65.35%	69.79%	61.85%	55.28%
**Flesch–Kincaid grade level**	6.94	6.78	6.05	7.13	8.00
**Coh-Metrix L2 readability**	21.09	26.07	26.12	18.47	22.48
**Coh-Metrix narrativity**	27.8%	18.9%	16.1%	15.4%	8.5%
**Coh-Metrix syntactic simplicity**	91.8%	78.8%	93.1%	96.8%	98.3%
**Coh-Metrix word concreteness**	15.4%	74.5%	88.9%	34.5%	90.7%
**Coh-Metrix referential cohesion**	77.9%	87.1%	82.4%	83.4%	46.0%
**Coh-Metrix deep cohesion**	50.8%	5.7%	24.5%	21.5%	75.8%
**CELEX word frequency for content words, mean**	2.08	2.14	2.19	2.02	1.90
**CELEX log frequency for all words, mean**	2.85	2.89	2.72	2.94	2.61
**Age of acquisition for content words, mean**	376.61	344.25	322.08	410.22	340.03
**Familiarity for content words, mean**	564.62	575.67	562.33	548.81	554.20
**Concreteness for content words, mean**	352.18	406.40	410.86	390.21	431.22
**Imageability for content words, mean**	361.26	444.50	438.33	419.93	460.06

**Note.* Psycholinguistic variables of the text’s lexical properties (word frequency, word length,

from the English Lexicon Project (Balota et al., 2007), the Kuperman age-of-acquisition (AoA) database (Kuperman et al., 2012), the MRC Database (Coltheart, 1981), and the Brysbaert concreteness database (Brysbaert et al., 2014). Bootstrapped one-way analyses of variance (ANOVAs) revealed no significant difference between the average values across all five texts for the average number of syllables (NSyll, *F* = .05, *p* = .99), lexical decision time (LDT, *F* = 1.07, *p* = .38), log frequency (*F* = .25, *p* = .91), naming response time (NRT, *F* = 1.41, *p* = .23), orthographic neighborhood density (OLD, *F* = .04, *p* = .99), phonological neighborhood density (PLD, *F* = .34, *p* = .85), concreteness (*F* = .24, *p* = .91), and number of phonemes (NPhon, *F* = .02, *p* = .99). However, one-way ANOVAs for word length and AoA were significant at *p* < .05 (*F* = 3.27, *F* = 3.32, respectively), suggesting that the average length of words and age at which these words are acquired may differ across the five texts.

## Footnotes

1 The use of a single score for reading comprehension is less reliable as an index because reading skill consists of multiple components including speed and accuracy (Gough & Tunmer, 1986). So far, different criteria have been used to divide participants into subgroups according to their reading performance (with reading time being an important one), but there are no consistent and well-accepted single criteria.

2 In our network science approach, we can use the same repeated words (nodes) and multiple saccades between words (edges) to collectively estimate the connections among nodes and edges. In previous approaches (e.g., von der Malsburg & Vasishth, 2011), each fixation is regarded as a unit for estimating the reading patterns. See further discussion below.

3 It is important to note that one should not directly correlate the high-vs.-low “efficiency score” with the literal meaning of high-vs.-low efficiency: in this case, a lower global efficiency score actually reflects a more efficient processing strategy, as skilled readers tend to skip repetitive words and function words in deriving the meaning of the text.

4 The sentence-based presentation used in the current study was based on the consideration of a trade-off between freedom of eye movements and eye drifting, so that eye drifting can be better corrected and controlled for. The flexibility of moving across the whole paragraph could lead to severe eye-drifting issues.
